# A machine-learning method isolating changes in wrist kinematics that identify age-related changes in arm movement

**DOI:** 10.1038/s41598-024-60286-1

**Published:** 2024-04-29

**Authors:** Aditya Shanghavi, Daniel Larranaga, Rhutuja Patil, Elizabeth M. Frazier, Satyajit Ambike, Bradley S. Duerstock, Anne B. Sereno

**Affiliations:** 1 Weldon School of Biomedical Engineering, Purdue University, West Lafayette, USA; 2 Department of Psychological Sciences, Purdue University, West Lafayette, USA; 3 Department of Health and Kinesiology, Purdue University, West Lafayette, USA; 4 School of Industrial Engineering, Purdue University, West Lafayette, USA; 5 School of Medicine, Indiana University, Bloomington, USA

**Keywords:** Geriatrics, Biomedical engineering, Machine learning

## Abstract

Normal aging often results in an increase in physiological tremors and slowing of the movement of the hands, which can impair daily activities and quality of life. This study, using lightweight wearable non-invasive sensors, aimed to detect and identify age-related changes in wrist kinematics and response latency. Eighteen young (ages 18–20) and nine older (ages 49–57) adults performed two standard tasks with wearable inertial measurement units on their wrists. Frequency analysis revealed 5 kinematic variables distinguishing older from younger adults in a postural task, with best discrimination occurring in the 9–13 Hz range, agreeing with previously identified frequency range of age-related tremors, and achieving excellent classifier performance (0.86 AUROC score and 89% accuracy). In a second pronation–supination task, analysis of angular velocity in the roll axis identified a 71 ms delay in initiating arm movement in the older adults. This study demonstrates that an analysis of simple kinematic variables sampled at 100 Hz frequency with commercially available sensors is reliable, sensitive, and accurate at detecting age-related increases in physiological tremor and motor slowing. It remains to be seen if such sensitive methods may be accurate in distinguishing physiological tremors from tremors that occur in neurological diseases, such as Parkinson’s Disease.

## Introduction

Aging is accompanied by the progressive deterioration of physiological and psychological functions due to a combination of structural changes such as the decline in strength in muscle, joints, bones, etc.^1^ and/or neurological conditions such as brain atrophy^2^. These deteriorations can affect the performance of daily activities involving arm motion^3^ and result in increased tremors^4–7^ and slowing of voluntary movement^8,9^.

Tremor is defined as a rhythmic, involuntary movement of a body part^10^. Depending on its etiology tremor may present in the form of rest (limb fully supported, such as on the arms of a chair, against gravity), kinetic (during movement), and/or postural tremor (while holding a limb in a particular position against gravity)^11^. All humans have tremors of small magnitude and high frequency called physiological tremor (PT)^12^, which increase in severity with age^13–15^. However, tremors may also occur as a result of a number of other conditions, including drugs^16^ or neurological disorders such as essential tremor (ET) or Parkinson’s Disease (PD)^3^. PT presents in the form of postural and/or kinetic tremor^12^; however, in our study, we have focused on postural PT, which may be helpful not only in identifying changes in aging but also in distinguishing hard to differentiate tremor symptoms in movement disorders such as PD versus ET or PD versus advanced physiologic tremor (e.g., reviews by Lenka et al.^3^ and Deuschl^17^).

Tremors generally have relatively constant frequency and variable amplitude (as reviewed by Wyne^18^), hence frequency domain analysis might lead to a more reliable detection and characterization of tremor than a time domain analysis. Previous research has found differences in ranges of tremor frequencies across conditions, for example with PT frequency in the 8–12 Hz range, PD tremor frequency in the 3–8 Hz band, and ET frequency spanning the 4–12 Hz range^5^.

Currently, the most reliable diagnostic tests for tremors are based on clinical scoring systems^19^. These scoring systems contain a substantial amount of error^20^ due to their inherent subjectivity^21^. In the past several years, a push towards more objective measures of tremors has been made. Numerous studies have analyzed the frequency domain of sensor signals for reliable and accurate measures of tremor^22–27^. For example, using accelerometer and electromyograph recordings of short 3-s windows in ET and PD patients, Luft and colleagues observed excellent classifier ($\sim$ 91% accuracy) in categorizing short 3-s time windows as either tremor or non-tremor^28^. Using only acceleration data from inertial measurement unit (IMU) sensors, Ali and colleagues^29^, have been successful in distinguishing between ET participants and healthy control. This study also found that the sensors located on the forearm had the best classification results. To the best of our knowledge, we are the first to use accelerometer and gyroscope data from non-invasive, wrist-worn IMU sensors to investigate subtle healthy age-related changes in PT.

Many studies, such as by Ali and colleagues^29^, have successfully used machine learning techniques (ML) techniques to help identify characteristics of tremor in different human diseases (see review by De et al.^19^). Although there are numerous applicable ML techniques, Support Vector Machine (SVM) is one of the most efficient machine learning algorithms^30^. While ML techniques like SVM can handle large numbers of variables, feature selection is crucial in ML because it improves model performance by reducing overfitting, enhancing model interpretability, and contributing to computational efficiency. The Minimal Redundancy Maximal Relevance (MRMR) algorithm first introduced by Peng et al.^31^ is a powerful algorithm that identifies a subset of input features (independent variables) by simultaneously maximizing relevance to the target variable and minimizing redundancy among selected features. Other feature selection methods in ML studies have used standalone methods such as analysis of variance^32–37^ or a two-step process combining the MRMR algorithm with secondary techniques such as with Fischer scores^38^, kernel canonical correlation analysis^39^ and kernel principal component analysis^40^. However, we are not aware of any studies that have used the MRMR algorithm followed by a variance threshold as a feature selection technique for an SVM to distinguish PT in young and older adults.

Age-related slowness of movement has also been examined in various studies to track age-related decline. In one such study, recording, and analyzing surface electromyographic waveforms from biceps brachii (agonist) and pronator teres (antagonist) muscles, Lewis and Brown^8^ found an age-related increase in motor response time (RT) to an auditory cue in the elderly. In a longitudinal study of aging, Fozard et al.^41^ found a slowing of simple RT to a single cue and a relatively greater slowing of RT to multiple randomized cues (disjunctive RT) across decades. Their study indicated consistent slowing and increased variability of reaction time to auditory cues with age. Other sensor-based studies have found increased response time with aging to various types of cues^42–46^. We investigated if commercially available lightweight sensors could be used not only to detect age-related increases in tremor but also slowing of voluntary arm movement in older participants, in order to capture a more complete picture of age-related changes in arm motion.

In summary, the overall goal of our study was to determine whether affordable, non-invasive IMU sensors could not only detect and identify changes in age-related arm motion changes in tremor and voluntary movement initiation but also achieve excellent classifier performance. The classifier performance serves to validate the efficacy of the set of selected motion signals in distinguishing age-related changes in tremors.

Given previous findings that sensors located on the forearm had the best signals for tremor classification^29^, we mounted our IMUs on the wrists. Using a power spectral density analysis, we transformed the 12 kinematic variables from the time domain to obtain the distributions of power in the frequency spectrum. Additionally, to optimize the identification of distinguishing tremor characteristics, we used a two-step process for feature selection (MRMR and analysis of variance) and then used the selected features in an SVM ML technique. Our goals were: (1) to identify which kinematic features show the most discriminating power between the two subject populations for changes in tremor; (2) to measure how well the ML classifier performed to classify the tremor of younger and older subjects; and (3) to test if the non-invasive wrist sensors could also reliably detect RT slowing in an older adult population.

## Results

### Analysis of tremor (Postural task)

#### Feature selection

**Figure 1:**
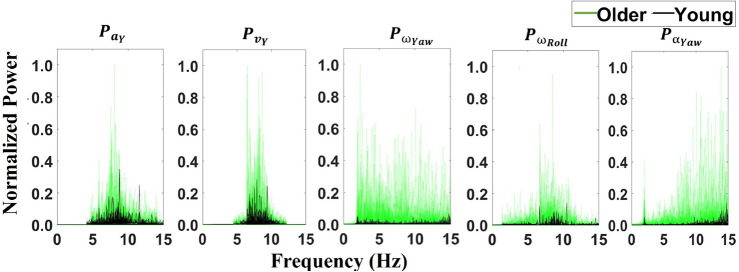
Features Selected for Classification. From features selected after the MRMR algorithm, use features that explain $\sim$ 90% of variance. Five features selected were Total Power of (1) linear acceleration in Y-axis ($P_{a_y}$), (2) linear velocity in Y-axis ($P_{v_y}$), (3) angular velocity in Yaw-axis ($P_{\omega _{Yaw}}$), (4) angular velocity in Roll-axis ($P_{\omega _{Roll}}$) and (5) angular acceleration in Yaw-axis ($P_{\alpha _{Yaw}}$). The variance explained by each variable was 27%, 23%, 16%, 15% and 10% respectively.

The MRMR algorithm, followed by the selection of features that accounted for at least 90% of the variance^47–49^, resulted in a final set of 5 features: (1) linear acceleration, Y-axis and (2) linear velocity, Y-axis, (3) angular velocity, Yaw-axis (4) angular velocity, Roll-axis, and (5) angular acceleration, Yaw-axis (See Table 1 and Fig 1). Physiologically these features indicate tremors in elbow flexion/extension (linear acceleration and linear velocity in Y-axis), elbow pronation/supination (angular velocity in Roll-axis) and wrist adduction/abduction (angular velocity and acceleration in Yaw-axis). The variance explained by each variable was 29.1%, 22.3%, 16.1%, 15.4%, and 9.1% respectively.

**Table 1:** Variance explained.

Kinematic feature	Variance explained (%)
**Linear acceleration, Y-axis** **(**$\bf P_{a_{Y}}$**)**	**26.8**
**Linear velocity, Y-axis** ($\bf P_{v_{Y}}$**)**	**23.1**
**Angular velocity, Yaw-axis** **(**$\bf P_{\omega _{Yaw}}$**)**	**15.9**
**Angular velocity, Roll-axis** **(**$\bf P_{\omega _{Roll}}$**)**	**15.1**
**Angular acceleration, Yaw-axis** **(**$\bf P_{\alpha _{Yaw}}$**)**	**10.1**
Angular acceleration, Roll-axis ($P_{\alpha _{Roll}}$)	2.9
Angular acceleration, X-axis ($P_{a_{X}}$)	2.5
Angular velocity, Pitch-axis ($P_{\omega _{Pitch}}$)	1.8
Angular acceleration, Pitch-axis ($P_{\alpha _{Pitch}}$)	0.9
Linear velocity, X-axis ($P_{v_{X}}$)	0.9
Linear acceleration, Z-axis ($P_{a_{Z}}$)	0.7
Linear velocity, Z-axis ($P_{v_{Z}}$)	0.2

Table shows the mean variance explained by each kinematic feature used in the feature selection (over a 1000 epochs). Selected features that explain approximately 90% variance are highlighted in bold.

#### Classification

**Figure 2:**
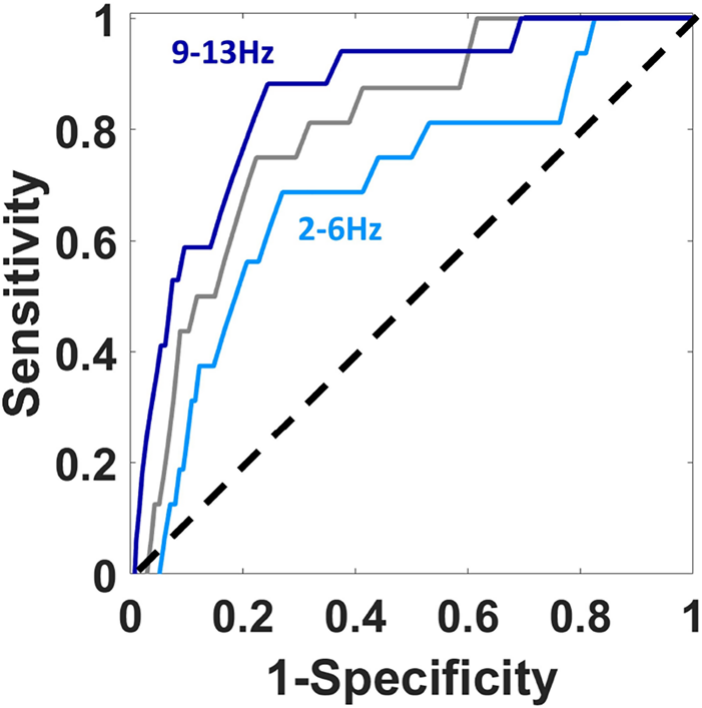
Performance of SVM Classifier. AUROC curves for the entire frequency range i.e. 2–15 Hz (grey), frequency range with best performance (dark blue), and frequency range with worst performance (light blue). Best performance was seen at 9–13 Hz (AUROC = 0.86) and worst performance was seen at 2-6Hz (AUROC = 0.65).

Using the 5 selected features as predictors in an SVM classifier, we tested which frequency ranges of these features provide the best classification performance (see Table 2). The best AUROC performance (0.86) and accuracy performance (89%) was seen in the 9–13 Hz range, a range that is very close to the range of PT identified in past literature (8–12 Hz^5^). The worst performance was seen in the lower frequency ranges, 2–6 Hz range for AUROC (0.65) and 4.5–8.5 for accuracy (55%), see Fig 2 and Table 2.

**Table 2:** AUROC scores and accuracy for all frequencies analyzed for classification performance.

Frequency range (in Hz)	AUROC score	Accuracy (%)
2–6	0.65	81
2.5–6.5	0.78	81
3–7	0.78	81
3.5–7.5	0.76	71
4–8	0.81	62
4.5–8.5	0.81	54
5–9	0.80	62
5.5–9.5	0.81	71
6–10	0.81	71
6.5–10.5	0.83	71
7–11	0.83	81
7.5–11.5	0.83	86
8––12	0.83	87
8.5–12.5	0.83	89
**9–13**	**0.86**	**89**
9.5–13.5	0.84	88
10–14	0.83	81
10.5–14.5	0.74	81
11–15	0.69	81
2–15	0.73	81

The 9–13 Hz window (in bold) shows the best performance, with the highest AUROC score (0.86) and highest accuracy (89%), while lower frequencies, including 2–6 Hz, show the worst AUROC performance (0.65) and 4.5–8.5 Hz the worst accuracy (54%).

#### Model validation

**Figure 3:**
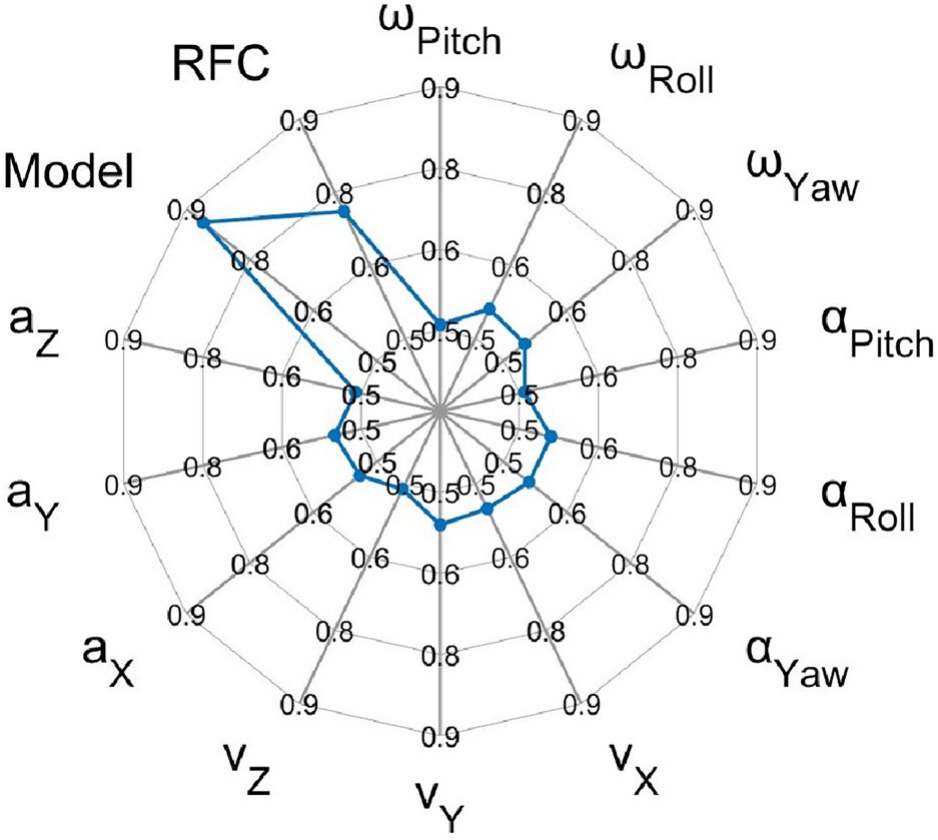
Spider plot of mean AUROC scores of 1000 epochs. The scores for each kinematic feature, the Model, and the average score for the 791 other possible random combinations of 5 features (RFC) in the 9–13 Hz frequency range are shown. The Model in the figure is the SVM classifier with 5 features selected based on the algorithm described in section “Feature selection”. The Model has the highest mean AUROC score.

As seen in Fig. 3, our model was able to predict group membership best, in the 9–13 Hz frequency range, as indicated by the highest mean AUROC score (0.86) when compared to the performance of any individual feature (highest mean AUROC score = 0.51) as well as to the average performance of all other 791 randomly selected 5 features (.77).

### Analysis of Response Time (Pronation–Supination task)

**Figure 4:**
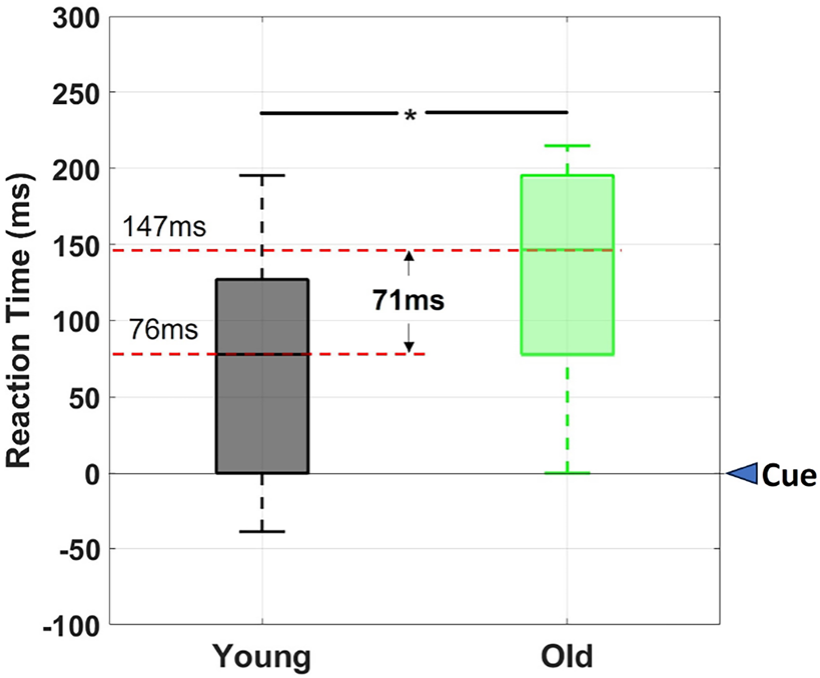
Boxplot of response times of younger and older adults. Cue was the word “Start”. Mean response time for younger adults was 76 ms and 147 ms for older adults ($p= 0.016$, w $= -3.765$, $\eta ^{2}= 0.5124$).

Figure 4 shows that older adults had a significant slowing of RT (147 ms) compared to younger adults (76 ms) (71 ms difference, Wilcoxon ranked-sign test; W = $-3.765$, $p= 0.016$, $\eta ^{2}= 0.5124$).

## Discussion

Using lightweight, commercially available sensors, we demonstrate here a method that achieves excellent classification performance of tremors in younger versus older adults as well as a method to quantify motor slowing in the initiation of arm movements. For tremors, our research identified five wrist kinematic variables in a postural task that optimally distinguish tremors in older adults from their younger counterparts. These selected features exhibit peak classifier performance in the 9–13 Hz range, matching closely with previously identified^5^ wrist PT frequency ranges. By using a combination of these selected features our model classifier predicting group membership achieves excellent performance and outperforms models using only any one individual feature (mean AUROC = 0.86 vs. mean AUROC = 0.51, respectively). Additionally, these same sensors were also sensitive enough to detect a $\sim$ 71 ms slowing in voluntary arm movement initiation in older adults, agreeing with previous studies showing age-related slowing^50^. The methods reported herein may be useful in guiding future research aimed at distinguishing between different neurological disorders.

### Importance of feature selection

Several studies have explored the kinematic composition of tremors^27,51–54^. However, to the best of our knowledge, we are the first to employ a data-driven feature selection process to do so. Our method demonstrates the efficacy of employing feature selection techniques to discern the primary components of PT from a pool of 12 kinematic variables, shedding light on the crucial factors that distinctly contribute to the age-related changes in tremors.

Given that postural PT is a universal characteristic of adult humans^12^ but escalates in severity with age^13–15^, our results suggest that the distinct shift in postural PT composition severity with age is primarily observed in the Y-, Yaw-, and Roll-axes.

It is interesting to note that the Y-axis shows the most significance in the results of our feature selection. It suggests that, although we measured at the wrist, the tremor may originate in more proximal arm joints (such as the elbow or shoulder). In their analysis of postural tremors within their neurotypical older subject population Chan and colleagues^27^ found that tremors at the elbow were significantly more severe than at the wrist.

We also observed significant tremor components in the Roll-axis. Rahimi and colleagues documented motion measurements in ET and PD patients in three degrees of freedom^52^, a setup mapping to our Y-axis, Yaw-axis, and Roll-axis, and found tremor in the Roll-axis to be indicative of ET. As part of our protocol inclusion/exclusion criteria, our participants did not have a history of diagnosed ET. However, we know from the literature that ET frequency is in the 4–12 Hz range^5^ which has overlap with the range of PT frequency (i.e. 8–12 Hz^5^), and also tends to increase with age^55^. Chan and colleagues^27^ found that elbow pronation–supination (mapping to Roll-axis in our study) showed the biggest difference in severity in their comparison of tremor composition between PD patients with subclinical tremor and age-matched controls. These findings suggest a potential overlap in the tremor composition of of ET, PT and subclinical PD in the Roll-axis with differences in frequency range and severity. Our use of the MRMR algorithm for feature selection was a critical step to help identify the features with maximum relevance for distinguishing tremor differences between groups.

Our feature selection approach is generalizable and could be used to distinguish the changes in tremor composition that occur in healthy aging from pathological aging populations. Identifying the crucial component features distinguishing tremors arising from different pathophysiologies may aid in differential diagnosis, help track progression, and/or lead to more targeted medical interventions and assistive devices.

### Importance of frequency range

Using a data-driven approach, we identified that the 9–13 Hz frequency range best-differentiated tremors between our older and younger participant groups. Interestingly, this frequency range largely overlaps with wrist tremor frequencies labeled PT (8–12 Hz)^5^. However, some studies have also reported observing PT in the lower (1–4 Hz)^56^ and higher (15–30 Hz) ranges^57^. As PT frequency is affected by limb weight^58^, these alternative frequency ranges may be explained by the different placements of the sensors used in these studies. Further, IMU sensors suffer from low-frequency noise (< 4 Hz^59^) and, according to Chang and colleagues, there is a physiological limit to hand motion frequency somewhere within the 15–20 Hz range^60^. These effects suggest caution on the interpretation of differences found in either very low or high frequency^56,57^ results and necessitate further analysis. For this reason, many recent studies investigating arm movements, including our own study, bandpass their IMU signals, eliminating frequencies below 2 Hz^61^ and above 15 Hz^60,62^ to filter out noise from the sources mentioned above.

As briefly reviewed earlier, tremors may also occur as a result of other conditions, including ET and PD. However, previous research has suggested differences in the affected frequency ranges for these different tremor conditions. For example, changes in the 3–8 Hz range are associated with PD while changes in a broader range, 4–12 Hz, have been associated with tremors in ET patients^5^. It remains to be understood what the underlying physiological changes are that produce these frequency-specific changes (review by Hallett^63^). However, identifying such behavioral biomarkers is the first step in identifying, quantifying, and tracking changes. This baseline is also necessary to evaluate the effectiveness of interventions in reducing the severity of specific tremors, especially in patients who may have a combination of conditions that lead to tremors.

### Age-related motor slowing

Aging is associated with reductions in peripheral muscle strength and motor control (e.g., see reviews by^64,65^). However, high-resolution structural magnetic resonance imaging (MRI) shows that there is already cortical thinning by middle age with prominent atrophy in the primary motor cortex (e.g.,^66^; review by Clark and Taylor^67^), suggesting additional central functional (brain) deterioration with age. Further, some evidence suggests that as motor control declines in older adults, they become more reliant on cognitive control mechanisms^68^. Unfortunately, prefrontal structures that support cognitive control show the largest age-related decline^68^, potentially leading to further slowing and deficits in motor control. We found significant differences in RT (71 ms) between younger and older participants in a pronation–supination task using both a fixed and a relative threshold value for initiation of movement. Hence, such a simple behavioral biomarker may serve as a measure of cognitive control of voluntary movements and provide a simple yet sensitive tool to track these functional and difficult-to-measure age-related changes.

Studies in healthy younger adults have shown that the mean RT to temporally varied auditory stimuli is approximately 284 ms^50^ however our results show a much faster mean RT than this value (mean RT for younger adults = 76 ms and mean RT for older adults = 147 ms). These results are likely due to a combination of practice before task recording^69^ as well as motor anticipation, given the experimenter counted down from 3 prior to saying “Start”.

The younger participants also had significantly more “early starts” (i.e., initiation of movement before the experimenter said “Start”). We know from literature that there is primer muscle onset latency, i.e. slowness of activation of muscles in advance of reaction time movements, associated with aging^70^. Also, anticipatory movements require cognitive control^71^ which, as established before, has been shown to decline with age^44,72^. Thus, reduced motor readiness and impaired anticipatory movements likely led to the slow RT in the older population.

### Movement artifacts

It is possible that we achieved such an effective classifier differentiating between the younger and older participants (AUROC score = 0.86, Accuracy = 89%) because we sampled during a postural (no movement) task, in contrast to past studies such as the one conducted by Luft and colleagues^28^. Although PT is also observed in kinetic tremor^63^ in an overlapping frequency range (8–12 Hz)^73^, a postural task may mitigate motion artifacts. Chan and colleagues, in their analysis of tremor between PD patients and age-matched controls, also found that the postural task showed the best results in tremor differences^27^. It is possible that motion artifacts may lead to added complexity in the measured feature signals, subsequently affecting both classifier performance and interpretation of results. Future studies are needed to investigate whether the tremor classification performance between older and younger adults differs in tasks with and without movement.

### Limitations

One possible limitation is medication differences. That is, it is possible that the increase in tremors in the older population may have occurred as a result of increased medication in the elderly^16^. Previous reports suggest that antihistamines and antidepressants can potentially exacerbate tremors^74,75^. Two subjects in our older population were on antidepressant medication, with one of these subjects simultaneously taking antihistamines. Similarly, there were three subjects in our young adult group who were taking anti-depressant medication. To test if medication differences affected our findings, we re-analyzed our data after removing these 5 participants. We found similar findings. Namely, we found the same five wrist kinematic variables, which resulted in peak classifier performance in the 9–13 Hz range, with performance (AUROC of 0.88) closely matching the full data set (AUROC of 0.86). In addition, we found a similar slowing in voluntary arm movement initiation (72 ms vs. 71 ms for the full data set). Hence, medication differences did not alter our main findings.

Another possible limitation of our study is the unequal sample sizes. Unequal sample sizes in classification models can be a cause of bias and insufficient power. However, some have argued that a larger control (as opposed to experimental) group, such as we have, may lead to more power^76^. More importantly, some studies have shown that the AUROC^77^ and/or F1 score^78^ are acceptable metrics to evaluate the performance of imbalanced groups in classification. However, there is some debate regarding which one is the more robust metric for this scenario. Our results show a very high mean AUROC score. We also calculated a mean F1 score (average score over a 1000 epochs) for the classification model in the 9–13 Hz and also obtained a high performance metric (F1 score = 0.88). These metrics suggest our findings were reliable with both metrics, suggesting no bias.

In this study, we split all the observations between training and testing sets. However, given there were two observations (two wrists) per subject, there exists the possibility that different observations from the same participant were included in both training and testing sets. Having an observation from the same subject in both training and testing sets could have possibly biased the model (making model performance metrics more optimistic), due to possibly higher inter-subject variance and lower intra-subject variance. However, in a small, unbalanced sample size such as ours, it is also possible that restricting the model to fewer subjects in training (as would be needed to avoid the above issue) increases overfitting of the model to the training data by learning participant-specific patterns rather than a generalizable pattern (biasing model performance metrics to underperform). A future study with a larger, more balanced sample size is needed to better explore how performance of the algorithm changes by splitting the data so that all observations from a participant are either in training or testing sets but not both, similar to the study by Gholamiangonabadi and colleagues^79^.

Finally, it is possible that the RT analysis could have been biased by the experimenter purposely pressing the button for the “Start” event marker too early or fast for the Older participants, thus resulting in the appearance of a slower RT in this group. We used the countdown (i.e. “3.2.1.Start”) to decrease experimenter button press variability (as well as participant RT) based on findings in the literature that auditory rhythms decrease movement variability (e.g. Varlet et al.^80^). Further, if the experimenter was biased to go slower for older participants (delaying button press), this would decrease RT in Older participants, the opposite of what we found. Nonetheless, our design made it more difficult for any experimenter bias. Ideally, the stimulus (i.e. “Start”) and event marking would have been automated to occur synchronously. Unfortunately, the Consensys Pro software, which was used for data acquisition and event marking, did not have the capability of generating or recording an external sound or external event marker. A future study interested in more accurately capturing auditory-initiated response differences will have to use a different sensor system that allows for event marking of an auditory cue.

### Future directions

These findings suggest many possible exciting future directions, such as identifying features that distinguish healthy aging from other tremor disorders (with onsets in the aging population) or identifying universal features that can differentiate multiple disorders and healthy aging at the same time. Indeed, examination of kinematic biomarkers either across broader more evenly distributed ages or across repeated sampling (i.e., repeated sampling over short temporal intervals to detect changes) may also potentially provide key features to help identify and disentangle age-related from disease-related tremors. A larger number of trials per participant may also enable the study of the individuality of tremors in participants using an analysis similar to that by Kopnarski and colleagues^81^.

Since tremors are affected by limb weight^58^, it may be that the classifier would perform even better with similar kinematic features from a lighter appendage, like the fingers, or worse from a heavier appendage like the legs. However, past studies investigating physiological and sub-clinical PD tremor^27^ have interestingly found that inertial sensors measuring at the proximal joints of the arm show significantly more tremor than at the distal joints. Future studies using our method to analyze PT at different joints in the arm could shed more light on this debate.

The portability of IMUs could facilitate remote monitoring of tremor-related diseases, reducing the need for patients to travel to the hospital/clinic as frequently. Such portable IMUs may also make it possible to identify tremor episodes over extended periods. Wearable devices, such as Fitbit smartwatches, have already been adopted by many people. These devices carry similar inertial sensors^82^. Together these methods have great potential to enhance telehealth approaches to diagnosis and tracking interventions and inform the design of assistive devices for older adults. However, many challenges remain, such as capturing data offsite in an unsupervised setting.

## Methods

### Participants

Twenty-three younger (ages 18–20, 12 female) and nine older (ages 49–57, 7 female) adults participated in this study. Only two younger and one older adults were left-handed. Data from 5 younger subjects was not included due either to technical issues with data collection (3 subjects; sensor disconnect or software crash during data upload) or due to the subject not following instructions (2 subjects; moving repeatedly during the recording when instructed not to move). Hence, data from eighteen younger (7 female, age 18–20, mean = 19, SD = ± 1) and nine older (7 female, age 49–70, mean = 56, SD = ± 8) participants was used for analysis. Prior to providing informed consent, all of the older participants completed the University of California, San Diego Brief Assessment of Capacity to Consent (UBACC)^83^, to ensure there was no age-related cognitive decline diminishing their capacity to consent. All older participants were above the threshold (UBACC ≥ 15/20) and were considered to have adequate capacity to provide informed consent. All participants provided written informed consent approved by Purdue University’s Institutional Review Board (IRB-2021-179 for younger participants, and IRB-2022-1299 for older participants) and in accordance with the Declaration of Helsinki. Younger participants were recruited from, and received course credit (via SONA Systems), while older participants were recruited via flyers and were compensated $15 for their participation. Finally, older participants completed the Montreal Cognitive Assessment (MOCA) to screen for any mild cognitive impairment. All older participants were above the threshold (MOCA Score ≥ 23) indicating no mild cognitive impairment^84,85^.

**Figure 5:**
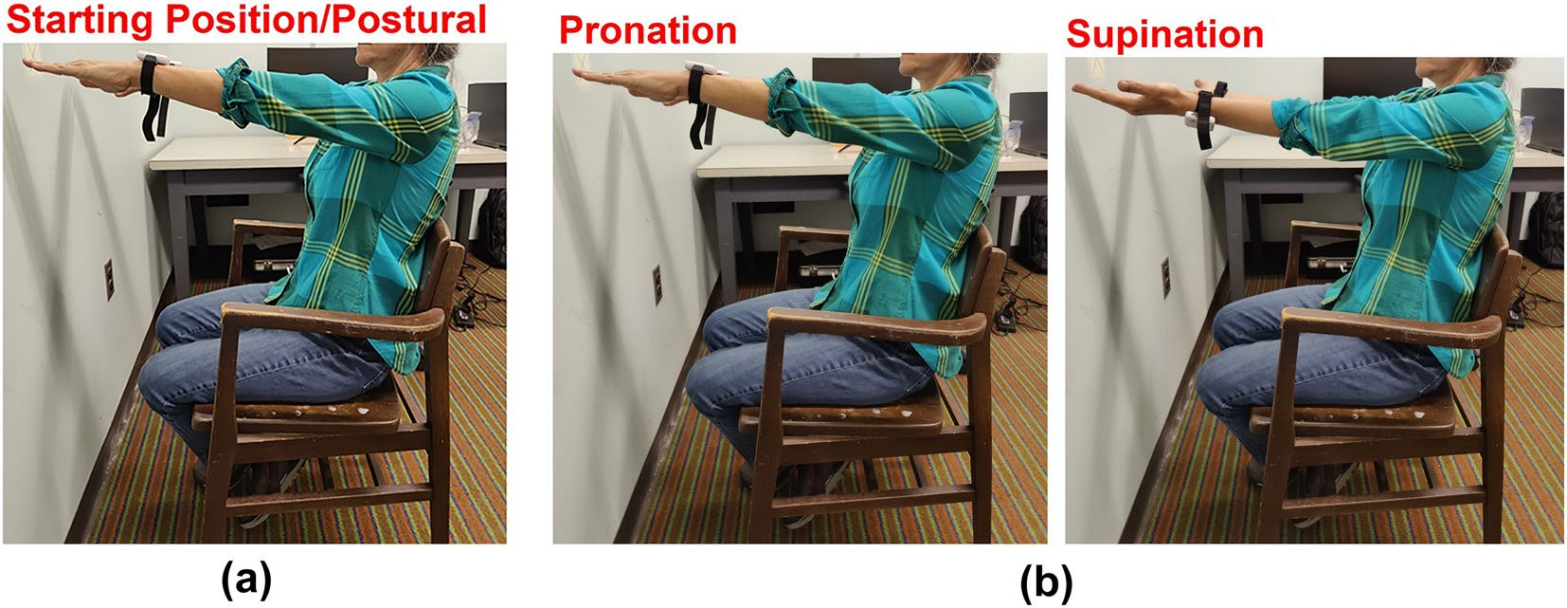
Behavioral tasks. (**a**) Postural task: Each participant was asked to stretch their arms out in front of the body with palms down. A 30-s recording was taken with the participant not moving while maintaining this position (**b**) pronation–supination task: Each participant was asked to extend their arms out in front of their body with the palms down and then after a warning signal (“3, 2, 1, Start”) to turn their palms up and down 20 times as fast and as fully as possible. The model in the figure is not a study participant and consent was obtained prior to the use of media.

### Measurement system

Two lightweight Shimmer3 IMU Units^86^ were attached to the wrists via elastic bands and recorded tri-axial linear acceleration (along X, Y, and Z axes in m/s^2^) and tri-axial angular velocity data (about the Roll, Pitch, and Yaw axes in deg/s). Sampling frequency was set to 100 Hz, consistent with previous tremor studies^5^, and sensors were paired with the ConsensysPRO^87^ software (on a laptop) via Bluetooth transmission to acquire movement signals and event markers (in ms) data.

**Figure 6:**
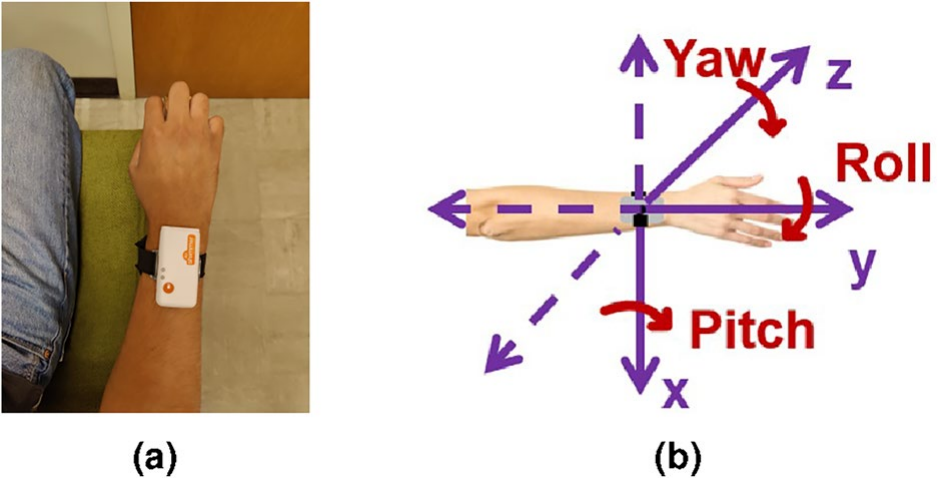
Sensor Setup. (**a**) Shimmer sensor secured to the participant’s wrist with elastic wristbands. (**b**) Shimmer Sensor Axes. Data was collected in the X, Y, Z, Roll, Pitch, and Yaw axes which were defined as shown. Physiologically these axes translate to elbow medial/lateral rotation, elbow flexion/extension, shoulder flexion/extension, elbow pronation/supination, wrist flexion-extension, and wrist adduction/abduction.

### Tasks

Each participant performed two tasks: A Postural task (see Fig. 5a) for analysis of tremor and a Pronation–Supination task (see Fig. 5b) for analysis of RT. In each task, we recorded one trial from each wrist, for a total of 2 observations per participant per task. Both these tasks are part of the clinical motor symptom assessment test for Parkinson’s Disease (MDS-UPDRS) and the official instructions were adapted^88^.

#### Starting position

For both tasks, the participant was first instructed to assume the *starting position*, with both arms stretched out in front of the body, keeping elbows at a 180°angle, forearms and wrists straight and parallel to the floor, with palms down and their back against the backrest and feet comfortable touching the floor (Fig. 5a).

#### Postural task

In this task, after assuming the starting position, the participant was then instructed to hold this position for 30-s without moving, until the experimenter said “Stop” (Fig. ﻿5a).

#### Pronation–supination task

In this task, after assuming the starting position, the participant was asked to turn the palm up and down alternately (referred to here as one repetition) 20 times as fast and as fully as possible while keeping the elbows at a 180°angle, forearms and wrists straight and parallel to the armrest, until the experimenter said “Stop” (Fig. 5b).

### Experiment procedure

After the assessment of capacity to consent (older subjects only), informed consent (all participants), and MOCA (older subjects only), a SHIMMER3 IMU was secured on the right and left wrist of the participant (as shown in Fig. 6a).

#### Sensor check

A sensor check test was first performed to ensure proper operation of the sensors for each arm and for each of the 6 axes (X, Y, Z, Roll, Pitch, and Yaw, defined as shown in Fig 6b).

#### Task order, instruction, and practice

After checking the sensors, all subjects performed the Postural task first and then the Pronation–Supination task. For each task, the participant was first given the instructions and procedures for the task. In the pronation–supination task, participants were told that after assuming the starting position, the participant should start only when they heard the experimenter say the words “Three. Two. One. Start” and stop each task when they heard the experimenter say “Stop”. Participants were then asked to assume the starting position and show the experimenter in order to demonstrate that they understood the instructions.

#### Recording, zeroing, start and stop

**Figure 7:**
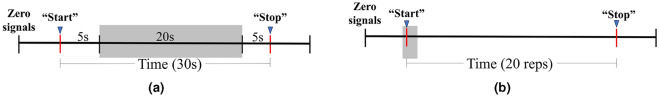
Task Timeline. (**a**) Postural Task (**b**) pronation–supination task. All signals were first set to zero (Zeroing). Shading indicates time period analyzed (**a**) and range of calculated RT onsets across participants (**b**). As shown in the figure the experimenter started the data collection after zeroing the signals (and before announcing “Start”) and ended data collection after announcing “Stop”.

For each task, after practice, each participant was asked to assume the starting position and to stay as still as possible. Then, the experimenter started the recording, zeroed the sensors (*Zero Signals*), and announced to the participant the warning (“3, 2, 1”) followed by the start (“Start”) signal. After completion of each task, the experimenter announced ”Stop” and ended the recording. When the experimenter announced the words “Start” and “Stop,” they simultaneously pressed a button, which created an event marker in the data file. The ConsensysPro software has a built-in event marker and timer, which were used to mark the start and end of data collection and the task start and end cues (simultaneously with the words “Start” and “Stop” as shown in Figs.  7a,b), as well as used to measure the 30-second task time.

### Pre-processing data

All the data processing was conducted using MATLAB R2022a.

#### Filter data

A 2–15 Hz band-pass filter was applied to the linear acceleration and angular velocity signals in both Postural Task and pronation–supination task data to remove low-frequency sensor drift and high-frequency noise.

#### Pre-processing, postural task data only

After filtering, the following steps were applied to the Postural Task data:

#### Calculate linear velocity

For each participant, the linear acceleration data (from “Zero signals” to “End” in Fig. 7a) was integrated to calculate linear velocity using the trapezoidal method,

1 $$\begin{aligned} v(t) = \int _{0}^{t_{i}} a(t)dt = \frac{1}{2}\sum _{n=1}^{i} (t_{n+1} - t_{n})[a(t_{n}) + a(t_{n+1})] \end{aligned}$$

where *v*(*t*) and *a*(*t*) are the linear velocity and acceleration, respectively, for the time range $t_{i}$. The initial velocity at the start of recording was assumed to be zero (i.e., $v(0) = 0$).

Numerically integrating accelerometer signals introduces a drift in the processed velocity signal^89^ and many methods have been proposed to eliminate this drift^90–96^. We used a widely accepted method suggested in Slack and Ma^97^, wherein we detrend the signal (using the MATLAB function “*detrend*”) by removing the best straight-fit line from the data. Supplementary Fig. S1a illustrates two example participants’ data in the Y-axis (older participant in green, younger in black) showing the numerical integration of the linear acceleration signal to calculate the linear velocity signal.

#### Calculate angular acceleration

The filtered angular velocity ($\omega$) signals from the “Start” to the “Stop” events were differentiated to obtain the corresponding angular accelerations ($\alpha$) along the roll, pitch, and yaw axes:

2 $$\begin{aligned} \alpha (t) = \frac{d\omega (t)}{dt} \end{aligned}$$

where $\alpha (t)$ is the angular acceleration ($\alpha$) and $\omega (t)$ is the angular velocity as a function of time *t*.

Supplementary Fig. S1b illustrates two example participants’ data in the Yaw-axis (older participant in green, younger in black) showing differentiation of the angular velocity signal to calculate the angular acceleration signal.

#### Calculate total power in power spectrum density

In order to identify differences in tremor, we converted the 12 kinematic variables (linear and angular velocities and accelerations) from the time domain to the frequency domain, as many previous studies have done^29,98–100^; then conducted a Power Spectral Density (PSD) analysis to obtain the distribution of power in the frequency spectrum. To avoid any initiation (“Start”) and termination (“Stop”) motion artifacts, we only analysed the middle 20-s period of the 30-s Postural task. For each of the 12 kinematic variables, we normalized the power spectra by dividing all values by the maximum power value observed. Supplementary Fig. S1c shows an example of PSD analysis of one of the kinematic variables (angular acceleration in Yaw-axis) across Older (in green) and Younger (in black) groups and nicely illustrates that group differences are much more apparent in the frequency spectrum. For each participant, the Total Power was calculated using Eq. 3 as follows:

3 $$\begin{aligned} P_{total} = \sum _{f=i}^{f=j} P(f) \end{aligned}$$

where *P*(*f*) is the power at a particular frequency (*f*) in the periodogram. The frequency window over which Total Power ($P_{total}$) is calculated is *i* to *j*. We used the entire frequency range (2–15 Hz) for feature selection but also evaluated narrower frequency ranges (in frequency windows of 4 Hz with an increment of 0.5 Hz as shown by the beige bar in Supplementary Fig. S1c for classification as further discussed below.

To test for differences in Total Power values between the signals from the left and right wrist in each group, we conducted a pairwise Wilcoxon rank-sign test and found that their medians were not significantly different (all *p* > 0.05; see Supplementary Tables S1, S2). This finding is in agreement with past literature that found bilateral symmetry in PT^101^. Hence we used both left and right wrist data as separate observations for analysis of tremor (section “Analysis of tremor (postural task)”) and RT (section “Data analysis response time (pronation–supination task)”).

### Analysis of tremor (Postural task)

Left and right wrists of each participant were considered as separate observations, so that our dataset consisted of n = 54 observations (36 observations for Younger and 18 for Older participant group). Each observation contained a label (0 = Younger participant; 1 = Older participant) and k = 12 kinematic variables (normalized total power values for each of the 12 kinematic variables across the entire frequency range). To evaluate which kinematic features (i.e., tremor signals) best distinguish between the Younger and Older populations we used binary classification. We used a three-step algorithm of (1) Data Splitting (2) Feature Selection and (3) SVM Classification. The algorithm was run for 1000 epochs.

#### Data splitting

We selected 80% of the observations in the dataset and assigned them to a training set and the remaining 20% to a testing set. Each epoch (1000 total) utilized a different randomly selected training data and testing data sample.

#### Feature selection

We used the Minimum Redundancy Maximum Relevance (MRMR) method (fscmrmr function in MATLAB) on the training set to reduce the number of features (from k, to k^′^, where k′ ≤ k) and identify the optimal set of kinematic features (normalized total power scores of the entire frequency range) to classify Younger and Older participants. This method indicates how well a specific feature (percent variance accounted for) predicts the outcome variable (i.e., whether a participant is a member of the Younger or Older group) while minimizing redundancy (i.e., minimizing the correlation of the kinematic variables)^31^. The average variance explained for each feature across all epochs is shown in Table 1. For each epoch, we selected a subset of features, k^′^, that exceeded 90% of the total variance in the outcome (i.e., we added variables until their sum exceeded 90%). In addition, for each epoch, we created reduced training and testing sets, including only the selected features, k^′^, for each observation. These reduced training and testing data sets were used in the SVM classification.

#### SVM classification

In each epoch, the reduced training dataset was used to train an SVM classifier model. The goal of the classifier was to discriminate between Younger and Older participants. SVM has been shown to be superior at binary classification^30,102,103^ compared to various other ML techniques used to identify tremor characteristics (see review by De et al.^19^) and has been extensively used in previous studies for similar applications (e.g., Ali and colleagues^29^). For each SVM model, we used a linear kernel, with auto hyperparameter optimization and standardized features (using the *fitcsvm* function in MATLAB) to train the model on the reduced training set. The reduced testing set was used to test the performance of the model on classifying unseen data.

We analyzed the performance of the classifier using the total frequency range (2–15 Hz) for these features as well as using 4 Hz frequency windows, in increments of 0.5 Hz, to determine if particular frequency ranges provided better classification. The 4 Hz interval was chosen because previous research examining physiological tremor (PT) as well as tremors in many movement disorders has found differences in tremor frequencies in smaller frequency ranges, such as 4 Hz. As we discuss in the introduction, prior literature has reported PT frequencies occur in the 8–12 Hz range and PD resting tremor in the 3.5–7.5 Hz range^5^, ranges that would be captured by shifting, in 0.5 Hz increments, a 4 Hz interval.

#### Evaluation metrics

True Positive (TP), True Negative (TN), False Positive (FP), and False Negative (FN) were used to calculate the Area Under the Receiver Operating Characteristic curve score (AUROC score) and accuracy, as metrics to evaluate the performance of the classifier. The AUROC score was obtained using the MATLAB function *perfcurve* and the accuracy (classification accuracy) was calculated using Eq. (4).

4 $$\begin{aligned} Accuracy = \frac{TP +TN}{TP + TN + FP + FN} \end{aligned}$$

Mean testing accuracy of 1000 epochs was used as the accuracy of the classification.

#### Model validation

We used a bootstrapped method to evaluate the model performance as described in the sections above to reduce the bias of a single model by averaging the predictions of multiple models trained on different samples (resampled 80% training and 20% testing in each epoch). Traditional cross-validation methods such as Leave-One-Out, K-Cross, and Holdout (See review by Yates and colleagues^104^) have indeed been used extensively in the literature and may have perhaps been computationally more efficient than our bootstrapped method. However, these methods are generally used to compare different models to find the one with the least error. In our study, we seek to evaluate the feature set not the particular classification model. Given our small sample size, we believe this method allows for more number of possible training and testing sets than Leave-One-Out, K-Cross, and Holdout methods leading to a more robust testing of the feature set as predictors.

To test if the performance (mean AUROC score over 1000 epochs) of our feature set, obtained from the method described in section “Feature selection”, is able to predict group membership (Older or Younger participant) better than that of any individual feature on its own or indeed any other 791 possible combinations of 5 features from the set of 12 features ($$\left( {\begin{array}{c}12\\ 5\end{array}}\right)$$ = 792 features) in the 9–13 Hz frequency range, a model validation procedure was performed with the SVM classifier, similar to the analysis conducted by Sotirakis et al.^105^. We compared the performance of our model to the performance of any individual feature as well as to the average performance of all possible combinations of 5 features in predicting group membership.

### Data analysis Response Time (Pronation–Supination task)

Angular velocity in the Roll axis was used to calculate movement latency in the pronation–supination task since the motion was significantly localized to that axis. $t_s$ was the time at which the task start event marker was pressed (“Start”). The initiation of motion was defined as the timepoint, $t_m$, at which instantaneous angular acceleration in the Roll axis exceeded 5% of peak value of $\frac{\Delta \omega _{Roll}}{\Delta t}$ motivated by the method used in Kita et al.^106^. Hence, response time (RT) was equal to $t_m - t_s$. As a result, any participants who started before the “Start” event marker had a negative RT value. We expected participants to anticipate the task cue signal and thus have a distribution of RTs near or shortly after the start event time.

## Conclusion

We have presented preliminary evidence of a method to detect age-related changes in wrist tremor and voluntary movement initiation latency. Our findings demonstrate that analyzing simple kinematic variables available in affordable, commercial sensors can detect and track these age-related arm motion changes. Our method was sufficiently sensitive to detect increased small physiological tremor-like wrist motions and slowed movement initiation times in healthy older individuals as compared to young adults. It remains to be seen if it can help improve the detection and evaluation of tremors in the clinic with various etiologies and whether further advances are needed for telehealth.

### Supplementary Information

Supplementary Information 1.

## Data Availability

The underlying code for this study and training/validation datasets are not publicly available since it is still being further analyzed but some parts may be made available to qualified researchers on reasonable request to the corresponding author.
