# Supplementary material for: A machine-learning method isolating changes in wrist kinematics that identify age-related changes in arm movement
Source: Sci Rep. 2024 Apr 29;14:9765. doi: 10.1038/s41598-024-60286-1 (PMC11059369; doi:10.1038/s41598-024-60286-1)
Supplement: Supplementary file 1 — Supplementary Information 1. [file 41598_2024_60286_MOESM1_ESM.pdf]

Kinematic Feature	W	p-value
$P_{\omega_{Pitch}}$	-0.174	0.862
$P_{\omega_{Roll}}$	-0.047	0.962
$P_{\omega_{Yaw}}$	-0.016	0.987
$P_{\alpha_{Pitch}}$	-0.206	0.837
$P_{a_{Roll}}$	-0.174	0.862
$P_{a_{Yaw}}$	-0.016	0.987
P_{v_X}	-0.427	0.669
P_{v_Y}	0.522	0.602
P_{v_Z}	0.237	0.812
P_{a_X}	-0.332	0.739
P_{a_Y}	0.648	0.517
P_{a_Z}	-0.111	0.912

Table S1: Comparison of total power value data between left and right wrist for Younger participants (Wilcox rank-sign test). None of the variables show any significant differences (p-value>0.05).

Kinematic Feature	W	p-value
$P_{\omega_{Pitch}}$	-0.004	0.931
$P_{\omega_{Roll}}$	-0.088	0.863
$P_{\omega_{Yaw}}$	-0.002	0.931
$P_{\alpha_{Pitch}}$	-0.004	0.863
$P_{a_{Roll}}$	-0.078	0.730
$P_{a_{Yaw}}$	-0.089	0.931
P_{v_X}	0.067	0.863
P_{v_Y}	-0.004	0.931
P_{v_Z}	0.177	0.863
P_{a_X}	-0.082	0.931
P_{a_Y}	0.353	0.730
P_{a_Z}	0.083	0.931

Table S2: Comparison of total power value data between left and right wrist for Older participants (Wilcox rank-sign test). None of the variables show any significant differences (p-value>0.05).

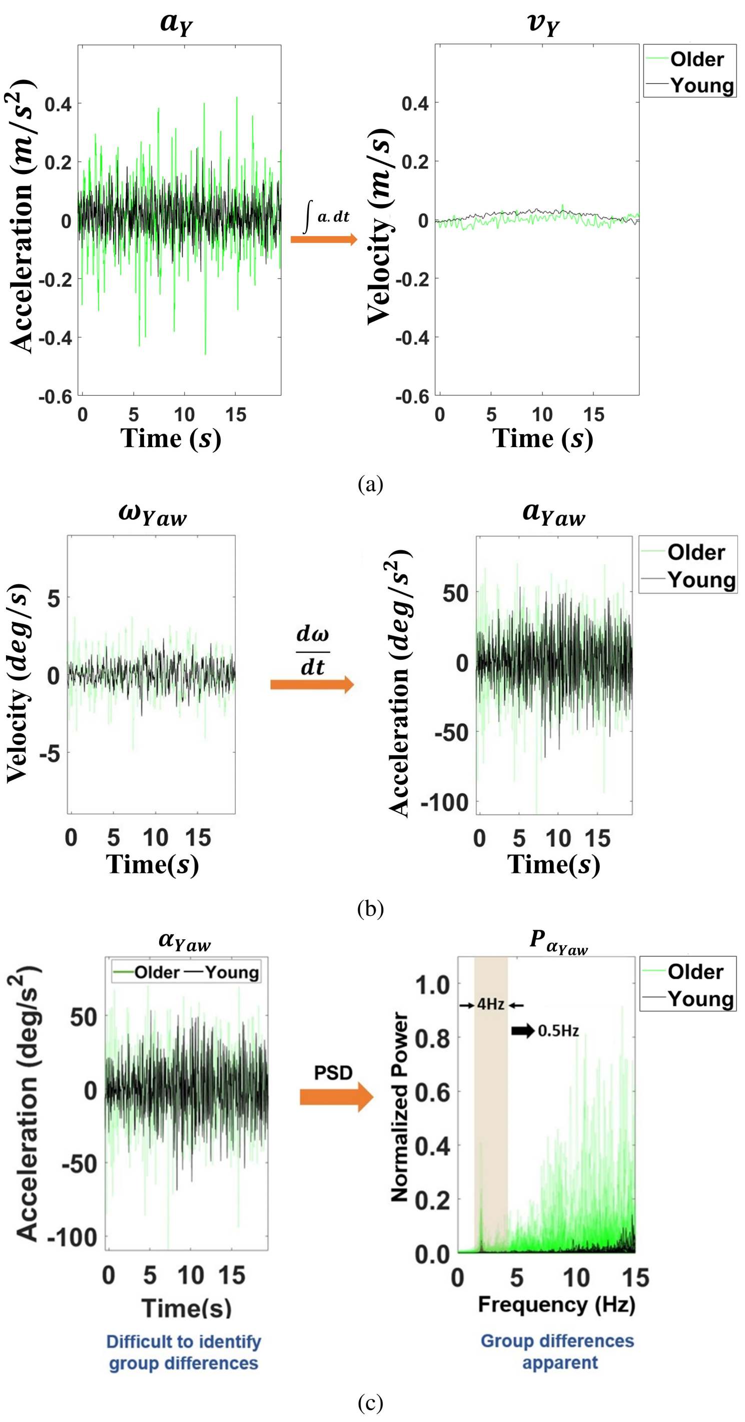

Figure S1: Preprocessing Steps. (a) Calculation of linear velocity (b) Calculation of angular acceleration (c) Power Spectral Density analysis. (a) and (b) show the raw signal from the sensor in the left panel and the calculated data in the right panel from a representative participant in the Older (in green) and Younger (in black) groups performing the Postural Task. (c) shows averaged group differences (Older in green, Younger in black) for an example kinematic variable (angular acceleration in the Yaw-axis) in the time domain (left panel) and normalized power in the frequency spectrum (right panel).
